# Commonly diagnosed mental disorders in a general hospital system

**DOI:** 10.1186/s13033-021-00484-w

**Published:** 2021-06-19

**Authors:** George Scott, Alessandra M. Beauchamp-Lebrón, Ashley A. Rosa-Jiménez, Javier G. Hernández-Justiniano, Axel Ramos-Lucca, Gloria Asencio-Toro, Julio Jiménez-Chávez

**Affiliations:** 1 Health Psychology Training and Research Core, School of Behavior and Brain Sciences, Ponce Health Sciences University, 388 Zona Industrial Reparada 2, Ponce, PR 00717 USA; 2 Department of Behavioral Medicine, Damas Hospital, 2213 Ponce Bypass, Ponce, PR 00717 USA

**Keywords:** Mental disorders, Depression, Neurocognitive disorder, General hospital, Health Psychology, Integrated care

## Abstract

**Background:**

Considering many patients receive care from general hospitals, these healthcare institutions are uniquely situated to address mental and physical health needs. Little is documented, however, on the common current mental disorders diagnosed in patients receiving care in general hospital settings, especially in Puerto Rico. The objective of this study was to characterize the five most common current DSM-5 mental disorder diagnoses made in patients receiving non-psychiatric medical and surgical care from a general hospital system in southern Puerto Rico between January 2015 and December 2019.

**Methods:**

Our clinical health psychology team provides integrated psychology consultation-liaison services to select clinical units in general hospitals across the southwestern region of Puerto Rico. The clinical team conducted routine standardized psychological evaluations at patients' bedside, arrived at a current DSM-5 diagnosis if warranted, and documented the diagnosis and other select variables. A retrospective study of cross-sectional data generated from the clinical team’s standardized evaluations of 5494 medical patients was implemented. Multinomial logistic regression analyses were used to assess the odds of being diagnosed with a current DSM-5 mental disorder during hospitalization.

**Results:**

Overall, 53% of the entire sample was diagnosed with a mental disorder during hospitalization. Major depressive, neurocognitive, anxiety, substance-related and schizophrenia-spectrum disorders were the most frequently diagnosed. Interestingly, females were 23% less likely to have been diagnosed with major depressive disorder than males (aOR: 0.769, CI [0.650, 0.909], p = 0.002). This is to say males evidenced 1.30 higher odds of being diagnosed with depression compared to their female counterpart. Age, biological sex, civil status, employment status, monthly household income, previous mental disorder and history substance use/abuse history was differentially associated with receiving a current DSM-5 disorder.

**Conclusion:**

The integration of clinical health psychology services within a general hospital facilitated our team’s work of identifying and treating co-occurring mental disorders among hospitalized patients receiving medical and surgical care. Future studies examining the opportunities and barriers of integrating clinical health psychology services within a general hospital’s administrative and clinical infrastructure for rapid identification and treatment of co-occurring mental disorders among medical patients is encouraged.

**Supplementary Information:**

The online version contains supplementary material available at 10.1186/s13033-021-00484-w.

## Introduction

Mental disorders are among the top causes of morbidity and account for approximately 14% of all deaths globally [45] and approximately 11% of the world’s population currently lives with a mental disorder [29]. Alarmingly, this estimate is higher in Puerto Rico, as revealed by findings from a recent behavioral health needs assessment conducted on the island. This assessment reported 12-month prevalence rates for any mental disorder to be approximately 24% among adults ages 18 to 64 [9]. Moreover, Puerto Ricans with a serious mental illness make up 7.3% of the adult population. It is estimated that nearly half (43.1%) of Puerto Rican adults with some type of mental disorder are in need of mental healthcare services [9]. These findings elucidate the magnitude of the mental health disparity crisis on the island, with inaccessible quality mental healthcare being a leading driver of such disparity [21, 43]. Since 2008, there has been a consistent migratory stream from the island to the United States. Included in this efflux of people are specialty mental healthcare providers such as clinical psychologists and psychiatrists, which further worsens the conundrum of lack of access to quality mental healthcare services [41, 42]. Thus, there is an urgent need to address this. Considering many patients receive medical and surgical care from general hospitals, these institutions are uniquely situated to facilitate access to mental healthcare services through rapid identification of mental disorders and initiation of appropriate interventions. In fact, a recent meta-analysis revealed that between 4 and 32% of inpatients receiving care in a non-psychiatric general hospital setting evidenced clinically significant levels of depression [46]. An earlier review of epidemiological studies revealed the prevalence of mental disorders in general hospital inpatients ranged from 41 to 46%, with organic brain syndrome, adjustment disorders with depressed mood and alcohol abuse being the three most commonly diagnosed [30]. Such a characterization has yet to be accomplished in Puerto Rico. Thus, the current study’s research team implemented a retrospective observational study of cross-sectional data to assess the most frequently diagnosed mental disorders in medical and surgical inpatients of a general hospital system in southern Puerto Rico between 2015 and 2019.

In order to achieve such a characterization, the systems that facilitate rapid identification of mental disorders and the provision of mental health services for medical and surgical inpatients of a general hospital are of critical value and worth briefly mentioning. Two widely used systems to integrate mental and physical health care of medical patients are the collaborative care [40] and consultation-liaison models [39]. The collaborative care model is mostly used in outpatient, primary-care settings and have limited implications for use in general hospital settings. The consultation-liaison (C-L) model, however, is more relevant for this present study’s aim and in fact, is the model which was used by our clinical team as detailed in the methods section below. Briefly, the C-L model entails two distinct yet overleaping components as the name implies: consultation and liaison. The consultation component is a process whereby attending physicians, surgeons, patients themselves or family members may request mental health services, and a clinical team member responds to this request by conducting an initial assessment, arriving at a clinical diagnostic impression, facilitating interventions as needed and making appropriate referrals if warranted. Liaison services are an especially crucial aspect in the provision of mental health care services to medical inpatients as this component requires a certain degree of integration at not only the service level (i.e., multidisciplinary teams) but at the organizational level as well [37]. Most attention in the literature, however, is given to the consultation aspect of the C-L model [14]. This selective attention is rather unfortunate as the liaison component, as overviewed in the discussion section below, is an aspect that warrants urgent attention and widespread implementation to achieve integrated care models in general hospital settings in an effort to curtail the mental health disparities that exist among medical populations such as in Puerto Rico. The success of detecting current mental disorders and facilitating interventions among the sample of medical and surgical inpatients of this present study is owed, in part, to the clinical health psychology C-L integration model implemented.

## Methods

The research team conducted a retrospective study examining cross-sectional biopsychosocial variables obtained from a sample of 5494 inpatients that received medical or surgical care from a general hospital in Ponce, Puerto Rico between January 2015 and December 2019.

### Participants

Participants were inpatients age 21 years and older, admitted to cardiovascular surgery, coronary care, intensive care and skilled nursing units of Damas Hospital, a general hospital in Ponce, Puerto Rico. These patients received a standardized clinical evaluation at bedside during their hospital stay and gave informed consent for data collection. Patients diagnosed with a current depressive, neurocognitive, anxiety, substance-related, and addictive and schizophrenia spectrum disorders according to DSM-5 criteria during their bedside standardized clinical evaluation were included in the study as these diagnostic categories were the most commonly diagnosed during this four-year period. Additionally, those who did not meet DSM-5 criteria for a current diagnosis during hospitalization were included as a comparison group. The Institutional Review Board of the Ponce Research Institute approved this study. Every author certifies that the study was performed in accordance with the ethical standards, as set by the 1964 Declaration of Helsinki and its later amendments.

### Procedures

#### Clinical team

The Clinical Psychology Services Program (CPSP) of Ponce Health Sciences University provides clinical health psychology services that include screenings, standardized clinical evaluations, brief psychotherapy, psychophysiological interventions, neuropsychological rehabilitation, and consultation-liaison (C-L) services in general hospital settings across the southern region of Puerto Rico. The provision of C-L health psychology services at Damas Hospital is the product of an academic agreement between Ponce Health Sciences University and Damas Hospital, an agreement that has been ongoing since 2002. The clinical team is coordinated by a psychiatrist and is made up of three licensed clinical psychologists, two of which are health psychologists, and one is a certified neuropsychologist. Additionally, 12 advanced practicum students of the PhD and PsyD clinical psychology programs of Ponce Health Sciences University are part of the clinical team and receive on-site, same day supervision for each patient seen and standardized clinical interview performed. The CPSP have established agreements with affiliate hospitals where standing orders are in place for select hospital units. Standing orders are the backbone of the clinical team’s liaison services and entail the administration of routine standardized clinical evaluations for all patients admitted to the following units: (a) cardiovascular surgery, (b) coronary care, (c) intensive care, and (d) skilled nursing units. Additionally, the clinical team provided clinical health psychology consults as requested by attending physicians, regardless of the inpatient unit to which the patient was admitted. For this present study, the research team analyzed sociodemographic and psychosocial data, current ICD-10 medical diagnoses, and current DSM-5 mental disorder diagnoses the CPSP clinical team made at patients’ bedside during the standardized clinical evaluation.

#### Standardized clinical evaluation

The standardized clinical evaluation entailed a mental status examination, exploration of psychological and emotional symptoms, a review of past physical and mental illness history, and a review of social functioning. Clinical inventories such as the Mini Mental Status Exam [15], Beck Depression Inventroy-II [6], and Beck Anxiety Inventory [7] were used to aid in the exploration of mental status and psychological-emotional symptomology. Additionally, a review of laboratory results, medications, and physician and nurse progress notes was conducted to aid in ruling out organic causes of possible mental aberrations. The CPSP clinical team performed the standardized clinical evaluation at the patient’s bedside. If warranted, the clinical team made a current DSM-5 diagnosis and a corresponding brief treatment plan was implemented for the duration of the patient’s hospital stay. For patients that did not evidence clinically significant symptoms as determined by the DSM-5 during evaluation, no diagnosis was made, and these patients’ data was used as a comparison group. The clinical inventories mentioned above were not included in the data analysis process as these measures were not available in the digital database alongside the current DSM-5 diagnoses and sociodemographic variables.

### Measures

#### Sociodemographic, medical diagnosis, and past mental health history

Questionnaires generated by the clinical team were used to collect the following biopsychosocial variables: age, biological sex, civil status, level of education, employment status, and monthly household income. Past history of mental disorder and history of substance use/abuse variables were also included in the study. ICD-10 medical diagnoses were obtained from chart reviews performed by the CPSP clinical team.

### Data analysis

SPSS 27 (IBM Corp) was used to perform data analysis. Data preparation and exploration procedures were conducted, and data was examined for errors and quality. Missing data analysis was conducted, and missing variables were replaced with multiple imputation linear regression method, with 10 imputations utilized. [31]. All missing data (12—monthly household income variables and 23—civil status variables) in this dataset were less than 5%. Descriptive statistics were performed to assess measures of central tendencies, standard deviation, and confidence intervals. Chi-squared tests of independence were conducted to assess associations between the top five diagnosed mental disorders and select sociodemographic indicators. Multinomial logistic regressions were conducted to evaluate the odds of being diagnosed with a DSM-5 depressive, neurocognitive, anxiety, substance-related, or schizophrenia spectrum disorder compared to no disorder among inpatients. The Benjamini-Hockberg procedure [8] was applied to control for false discovery rates due to multiple comparisons in the adjusted multinomial logistic regression model.

## Results

Data from a total of 5494 inpatients was analyzed. The mean age of this patient sample was 62.2 years, 95% CI [61.75, 62.65]. There were more females (57.8%) than males and the majority of patients were unmarried (53.2%). Additionally, 83.6% were unemployed or retired and 26% reported having obtained less than a high school education. Overall, more than half (53%) of the entire sample was diagnosed with a current DSM-5 mental disorder during hospitalization. Major depressive disorders accounted for 42%, neurocognitive (33%), anxiety (11%), substance-related (8%), and schizophrenia spectrum (6%) of the five most frequently diagnoses made by the CPSP clinical team. Further, 97% of all patients evidenced a chronic physical condition. Ninety-nine per cent (99.4%) of male patients had a chronic physical condition compared to 95% of female patients. Fifty-nine percent (59%) of male patients had comorbid mental and physical conditions compared to 48% of females.

### Specific DSM-5 diagnoses

Among major depressive disorders diagnoses (MDD), recurrent episodes (70%) were most frequently diagnosed and recurrent episodes with psychotic characteristics (6.9%), the least frequent. Within neurocognitive disorders (NCD), delirium due to another medical condition (24.8%) was most commonly diagnosed and unspecified delirium (6.8%) the least common. Generalized anxiety disorder (72.5%) was the most frequently diagnosed anxiety disorder and alcohol use disorder (48%) the most frequently diagnosed substance-related disorder. Schizophrenia and brief psychotic disorder were the most and least common diagnosed within the schizophrenia spectrum disorders, 34.6% and 6.7%, respectively.

### Comorbid chronic physical conditions and diagnosed mental disorders

Overall, cardiovascular disease, orthopedic conditions, chronic respiratory, gastrointestinal and cancer diagnoses were the most prevalent comorbid medical conditions in this sample. Thirty-five percent (35%) of this sample were evaluated in the skilled nursing unit, 24% coronary care, 22% intensive care and 19% cardiovascular surgery unit. Among patients diagnosed with major depressive disorders, slightly more than one in five patients (24%) also lived with a co-occurring cardiovascular disease. The least frequent co-occurring chronic physical disease among patients with depressive disorders was immunologic disease, accounting for 0.2% of all ICD-10 diagnoses among this patient group. Chronic neurological diseases were the most common co-occurring physical conditions among patients diagnosed with a DSM-5 neurocognitive condition (22%), and chronic endocrine disorders (1%) were the least common. Slightly more than one out of every four patients (27%) diagnosed with an anxiety disorder also had a co-occurring cardiovascular disease. Chronic immunologic disorders were least common among this group of patients, accounting for 0.3% of ICD-10 diagnoses. Similarly, patients diagnosed with a substance-related disorder had higher cardiovascular disease comorbidity (18%) than any other chronic physical condition. Lastly, among patients diagnosed with a schizophrenia spectrum disorder, 5.8% also lived with a co-occurring cardiovascular disease.

Routine standardized clinical evaluations as part of the administrative standing orders accounted for 84% of all DSM-5 diagnoses made and the remaining 16% were made from physician or patient requested consults. The two most common diagnoses resulting from the administrative standing orders (routine standardized clinical interviews—the liaison component) were neurocognitive and major depressive disorders, accounting for 20% and 19%, respectively. Diagnoses made from all consults, however, consisted primarily of major depressive disorders (37%), followed by substance use disorders (9%).

### Association of biological sex and mental disorder diagnoses in a general hospital setting

An unadjusted multinomial logistic regression model revealed that biological sex is differentially associated with being diagnosed with a current mental disorder during hospitalization. The overall model was significant χ^2^ (5) = 215.659, p < 0.001, and biological sex accounted for 4.1% of the variance found within the mental disorder diagnosis categories (Nagelkerke pseudo R^2^ = 0.041). Overall, male patients had higher odds of being diagnosed with a current DSM-5 diagnosis compared to females. However, being diagnosed with an anxiety disorder was not significantly associated with biological sex. Female patients were 15.7% less likely to be diagnosed with MDD compared to male (OR: 0.842, 95% CI [0.732, 0.968], p = 0.01). This is to say males were 1.18 times more likely to have been diagnosed with a current MDD at bedside than females. Moreover, males evidenced 1.78 higher odds of being diagnosed with a form of NCD compared to females (OR: 0.561, CI [0.484, 0.651], p < 0.001). Male patients also evidenced much higher odds (7.46) of being diagnosed with a substance related disorder compared to females (OR: 0.134, CI [0.094, 0.190], p < 0.001). Lastly, female patients were 49.5% less likely to be diagnosed with a schizophrenia spectrum disorder than males (OR: 0.505, CI [0.371, 0.688], p < 0.001). Stated differently, male patients evidenced 1.98 higher odds of being diagnosed with a schizophrenia spectrum disorder during hospitalization see Tables 1, 2, 3.

**Table 1:** Sociodemographic Characteristics (n = 5494)

Variable	Frequency	%
Age group (years)		
21–30	425	7.7
31–40	342	6.2
41–50	441	8
51–60	923	16.8
61–70	1416	25.8
71–80	1286	23.4
81–90	568	10.3
≥ 91	93	1.7
Biological sex		
Female	3175	57.8
Male	2319	42.2
Civil status		
Not married	2924	53.2
Married	2570	46.8
Employment status		
Not employed (including retired)	4593	83.6
Employed	901	16.4
Education level		
No formal education	93	1.7
< High school	1348	24.5
High school	2181	39.7
Postsecondary and beyond	1872	34.1
Monthly household income (USD)		
$0	120	2.2
≤ $300	612	11.1
$301–$900	2091	38.1
$901–$1500	1457	26.5
$1501–$2100	602	11
≥ $2101	612	11.1

**Table 2:** Profile of sociodemographic, previous mental disorder history and chronic co-morbid physical illness

(n = 5494)	Major depressive disorder (n = 1214)	Neurocognitive (n = 995)	Anxiety (n = 314)	Substance-related (n = 217)	Schizophrenia spectrum (n = 173)	No DSM-5 diagnosis (n = 2581)
Mean (95% CI)						
Age (years)	57.63 (56.68, 58.58)	73.41 (72.64, 74.17)	56.90 (54.94, 58.86)	51.71 (49.45, 53.97)	49.83 (47.23, 52.64)	62.30 (61.66, 62.94)
Frequency (% within mental disorder category)						
Biological sex						
Female	722 (59.5)	492 (49.4)	199 (63.4)	41 (18.9)	81 (46.8)	1640 (63.5)
Male	492 (40.5)	503 (50.6)	115 (36.6)	176 (81.1)	92 (53.2)	941 (36.5)
Civil status						
Not married	734 (60.5)	521 (52.4)	164 (52.2)	153 (70.5)	135 (78.0)	1217 (47.2)
Married	480 (39.5)	474 (47.6)	150 (47.8)	64 (29.5)	38 (22.0)	1364 (52.8)
Education level						
No formal education	17 (1.4)	27 (2.7)	4 (1.3)	2 (0.9)	2 (1.2)	41 (1.6)
Less than high school	268 (22.1)	323 (32.5)	62 (19.7)	55 (25.3)	48 (27.7)	592 (22.9)
High school	508 (41.8)	375 (37.7)	123 (39.2)	111 (51.2)	81 (46.8)	983 (38.1)
Postsecondary and beyond	421 (34.7)	270 (27.1)	125 (39.8)	49 (22.6)	42 (24.3)	965 (37.4)
Employment status						
Not employed	1044 (86.0)	921 (92.6)	234 (74.5)	168 (77.4)	159 (91.9)	2067 (80.1)
Employed	170 (14.0)	74 (7.4)	80 (25.5)	49 (22.6)	14 (8.1)	514 (19.9)
Monthly household income						
≤ $300	221 (18.2)	130 (13.1)	29 (9.2)	54 (24.9)	48 (27.7)	250 (9.7)
$301–$900	462 (38.1)	403 (40.5)	131 (41.7)	82 (37.8)	83 (48.0)	930 (36.0)
$901–$1500	310 (25.5)	269 (27.0)	79 (25.2)	46 (21.2)	27 (15.6)	726 (28.1)
$1501–$2100	107 (8.8)	96 (9.6)	35 (11.1)	20 (9.2)	8 (4.6)	336 (13.0)
≥ $2100	114 (9.4)	97 (9.7)	40 (12.7)	15 (6.9)	7 (4.0)	339 (13.1)
History of substance use/abuse						
Positive history	391 (32.2)	238 (23.9)	87 (27.7)	196 (90.3)	68 (39.3)	568 (22.0)
Negative history	823 (67.8)	757 (76.1)	227 (72.3)	21 (9.7)	105 (60.7)	2013 (78.0)
Previous mental disorder						
Recurring mental disorder	774 (63.8)	225 (22.6)	159 (50.6)	102 (47.0)	152 (87.9)	459 (17.8)
No recurring mental disorder	440 (36.2)	770 (77.4)	155 (49.4)	115 (53.0)	21 (12.1)	2122 (82.2)
Chronic co-morbid physical illness						
Cardiovascular Disease	291 (24.0)	198 (19.9)	84 (26.8)	40 (18.4)	10 (5.8)	689 (26.7)
Orthopedic	77 (6.3)	110 (11.1)	34 (10.8)	16 (7.4)	1 (0.6)	485 (18.8)
Respiratory	96 (7.9)	110 (11.1)	34 (10.8)	9 (4.1)	10 (5.8)	123 (4.8)
Gastrointestinal	74 (6.1)	76 (7.6)	22 (7.0)	17 (7.8)	8 (4.6)	155 (6.0)
Cancer (unspecified)	38 (3.1)	17 (1.7)	10 (3.2)	6 (2.8)	2 (1.2)	146 (5.7)
Other	632 (52.1)	482 (48.4)	129 (41.1)	128 (59.0)	142 (82.0)	830 (32.2)
No diagnosis	6 (0.5)	2 (0.2)	1 (0.3)	1 (0.5)	0	153 (5.9)

**Table 3:** Specific DSM-5 diagnoses, consultation and liaison services

	Frequency	% (within DSM-5 category)
Major depressive disorders (MDD) (n = 1214)		
MDD, recurrent episode	855	70.4
MDD, single episode	122	10.1
MDD, recurrent with psychotic characteristics	84	6.9
Other	153	12.6
Neurocognitive disorders (NCD) (n = 995)		
Delirium due to another medical condition	247	24.7
Unspecified NCD	148	14.8
Delirium due to multiple etiologies	92	9.3
Mild NCD due to vascular disease	80	8.0
Unspecified delirium	68	6.8
Other	360	36.2
Anxiety disorders (n = 314)		
Generalized anxiety disorder (GAD)	228	72.5
Panic disorder	54	17.2
Other	32	10.1
Substance-related (n = 217)		
Alcohol use disorder	104	48.0
Tobacco use disorder	93	42.8
Other	20	9.2
Schizophrenia spectrum (n = 173)		
Schizophrenia	60	34.6
Unspecified schizophrenia spectrum and other psychotic disorder	36	20.7
Brief psychotic disorder	11	6.7
Other	66	38.0
Type of service		
Consultation	856	16
Standing order	4638	84

^**Other**^^**: t**^^**hese are DSM**^^**−**^^**5 diagnoses within the broader category mentioned. However, no single “other” diagnostic category was commonly diagnosed in this sample to warrant mentioning**^

### Adjusted multinomial model

The overall adjusted model was significant χ^2^ (70) = 2463.171, p < 0.001, with covariate variables accounting for 38.5% of the variance found within the DSM-5 diagnosis categories (Nagelkerke pseudo R^2^ = 0.385). Reference Table 4 for adjusted multinomial logistic regression values.

**Table 4:** Adjusted multinomial logistic regression

	B	S.E	Wald χ^2^	*p*	OR	95% Confidence interval	
						Upper	Lower
Major depressive disorders							
Intercept	− 1.724	0.211	66.951	< 0.001			
Age (years)	− 0.010	0.003	14.326	< 0.001	0.99	0.98	0.99
Female^a^	− 0.263	0.086	9.431	0.002	0.76	0.65	0.909
Unmarried^b^	0.346	0.080	18.617	< 0.001	1.41	1.20	1.65
Not employed^c^	0.426	0.117	13.331	< 0.001	1.53	1.21	1.92
No formal education^d^	0.141	0.316	0.199	0.65	1.15	0.62	2.13
< High school^d^	0.098	0.111	0.783	0.37	1.10	0.88	1.37
High school^d^	0.069	0.092	0.562	0.453	1.07	0.89	1.28
≤ $300^e^	0.755	0.165	20.912	< 0.001	2.12	1.53	2.94
$301–$900^e^	0.313	0.140	4.964	0.02	1.36	1.03	1.79
$901–$1500^e^	0.211	0.142	2.209	0.13	1.23	0.93	1.63
$1501–$2100^e^	− 0.025	0.169	0.022	0.88	0.97	0.70	1.35
Previous mental disorder	2.032	0.082	620.783	< 0.001	7.62	6.50	8.95
Substance use/abuse history^g^	0.211	0.092	5.248	0.02	1.23	1.03	1.48
Neurocognitive disorders							
Intercept	− 5.177	0.279	344.717	< 0.001			
Age (years)	0.056	0.003	259.377	< 0.001	1.051	1.058	1.065
Female^a^	− 0.601	0.086	49.125	< 0.001	0.54	0.46	0.64
Unmarried^b^	0.239	0.082	8.504	0.004	1.27	1.08	1.49
Not employed^c^	0.247	0.145	2.886	0.08	1.28	0.96	1.70
No formal education^d^	0.162	0.275	0.349	0.55	1.17	0.68	2.01
< High school^d^	0.134	0.108	1.544	0.21	1.14	0.92	1.41
High school^d^	0.103	0.099	1.086	0.29	1.10	0.91	1.34
≤ $300^e^	0.563	0.174	10.511	0.001	1.75	1.25	2.46
$301–$900^e^	0.240	0.144	2.791	0.09	1.27	0.95	1.68
$901–$1500^e^	0.100	0.146	0.470	0.49	1.10	0.83	1.47
$1501–$2100	− 0.028	0.173	0.027	0.87	0.97	0.69	1.36
Previous mental disorder	0.646	0.098	43.326	< 0.001	1.90	1.57	2.31
Substance use/abuse history^g^	− 0.070	0.098	0.510	0.47	0.933	0.770	1.12
Anxiety disorders							
Intercept	− 1.899	0.313	36.860	< 0.001			
Age (years)	− 0.012	0.004	8.235	0.004	0.989	0.981	0.997
Female^a^	− 0.088	0.138	0.410	0.52	0.91	0.69	1.19
Unmarried^b^	0.055	0.127	0.189	0.66	1.05	0.82	1.35
Not employed^c^	− 0.209	0.162	1.654	0.19	0.81	0.59	1.11
No formal education^d^	0.053	0.544	0.010	0.92	1.05	0.36	3.06
< High school^d^	0.052	0.182	0.082	0.77	1.05	0.73	1.50
High school^d^	0.026	0.145	0.032	0.85	1.02	0.77	1.36
≤ $300^e^	0.051	0.277	0.034	0.85	1.05	0.61	1.81
$301–$900^e^	0.304	0.208	2.131	0.14	1.35	0.90	2.03
$901–$1500^e^	0.033	0.214	0.023	0.87	1.03	0.67	1.57
$1501–$2100^e^	− 0.048	0.250	0.037	0.84	0.95	0.58	1.55
Previous mental disorder^f^	1.495	0.127	138.713	< 0.001	4.46	3.47	5.72
Substance use/abuse history^g^	0.154	0.148	1.083	0.29	1.16	0.873	1.55
Substance-related disorders							
Intercept	− 3.447	0.490	49.526	< 0.001			
Age (years)	− 0.029	0.005	31.886	< 0.001	0.97	0.96	0.98
Female^a^	− 1.317	0.197	44.755	< 0.001	0.26	0.18	0.39
Unmarried^b^	0.643	0.176	13.280	< 0.001	1.90	1.34	2.68
Not employed^c^	− 0.001	0.204	0.000	0.99	0.99	0.67	1.49
No formal Education^d^	− 0.279	0.793	0.124	0.72	0.75	0.16	3.57
< High school^d^	0.356	0.235	2.294	0.13	1.42	0.90	2.26
High school^d^	0.387	0.197	3.879	0.04	1.47	1.00	2.16
≤ $300^e^	1.016	0.346	8.605	0.003	2.76	1.40	5.44
$301–$900^e^	0.584	0.314	3.471	0.06	1.79	0.97	3.31
$901–$1500^e^	0.417	0.326	1.631	0.20	1.51	0.80	2.87
$1501–$2100^e^	0.396	0.373	1.123	0.28	1.48	0.71	3.08
Previous mental disorder^f^	1.053	0.167	39.982	< 0.001	2.86	2.06	3.97
Substance use/abuse history^g^	2.832	0.246	132.657	< 0.001	16.98	10.48	27.49
Schizophrenia spectrum disorders							
Intercept	− 5.054	0.598	71.347	< 0.001			
Age (years)	− 0.028	0.005	27.363	< 0.001	0.97	0.96	0.98
Female^a^	− 0.929	0.181	26.251	< 0.001	0.39	0.27	0.56
Unmarried^b^	0.914	0.205	19.835	< 0.001	2.49	1.66	3.73
Not employed^c^	0.959	0.311	9.510	0.002	2.60	1.41	4.79
No formal Education^d^	0.512	0.781	0.430	0.51	1.66	0.36	7.71
< High school^d^	0.758	0.248	9.331	0.002	2.13	1.31	3.47
High school^d^	0.400	0.213	3.531	0.06	1.49	0.98	2.26
≤ $300^e^	1.454	0.444	10.722	0.001	4.28	1.79	10.22
$301–$90^e^	1.107	0.419	6.983	0.008	3.02	1.33	6.87
$901–$1500^e^	0.382	0.444	0.738	0.39	1.46	0.61	3.49
$1501–$2100^e^	0.090	0.540	0.028	0.86	1.09	0.38	3.15
Previous mental disorder^f^	3.407	0.245	194.018	< 0.001	30.16	18.67	48.72
Substance use/abuse history^g^	0.032	0.189	0.029	0.86	1.033	0.712	1.49

Reference category for DSM-5 diagnoses is no current DSM-5 mental disorders

^a^Male is reference category

^b^Married is reference category

^c^Employed is reference category

^d^Postsecondary education and beyond is reference category

^e^ ≥ $2101 monthly household income is reference category

^f^No previous mental disorder history is reference category

^g^Negative substance use/abuse history is reference category

^*^p-values reported are those obtained before the Benjamini–Hochberg procedure was applied

#### Major depressive disorders

Overall, age, biological sex, civil status, employment status, monthly household income, previous mental disorder and substance use/abuse history was significantly associated with receiving a current DSM-5 major depressive disorder diagnosis. For each one-year increase in age, patients evidenced a 1% less chance of being diagnosed with a current MDD (OR: 0.990, CI [0.985, 0.995]). Females evidenced 23% lower odds of being diagnosed compared to males (OR: 0.769, CI [0.650, 0.909]). In other words, males had 1.30 higher odds of being diagnosed with a current depressive disorder. Compared to married patients, the ones who reported being unmarried were 1.41 times more likely to have been diagnosed (OR: 1.41, CI [1.20, 1.65]). Those who reported having a monthly household income of $ ≤ 300 had 2.12 higher odds of being diagnosed compared to patients reporting a household income ≥ $2101 per month (OR: 2.12, CI [1.53, 2.94]). Patients with previous mental disorders evidenced 7.62 higher odds of being diagnosed with a current depressive disorder compared to patients without a previously diagnosed mental disorder (OR: 7.62, CI [6.50, 8.95]). Lastly, patients with a past history of substance use/abuse evidenced 1.23 higher odds of being diagnosed with depression compared to those with no history of substance use/abuse (OR: 1.23, CI [1.03, 1.48]). Level of education was not significantly associated with being diagnosed with a current depressive disorder diagnosis at bedside. After adjusting for multiple comparisons, only age, biological sex, civil status, employment status, monthly household incomes of ≤ $300, and previous mental disorder remained significant covariates of being diagnosed with a current MDD.

#### Neurocognitive disorders

For every one-year increase in age, the odds of being diagnosed with a current neurocognitive disorder was relatively similar (OR: 1.058, CI [1.051, 1.065]). Female patients evidenced 45% lower odds of being diagnosed compared to males (OR; 0.54, CI [0.46, 0.64]). This is to say males had 1.82 higher odds than females of being diagnosed with a current neurocognitive disorder. Unmarried patients were 1.27 times more likely to have received a current diagnosis compared to married patients (OR: 1.27, CI [1.08, 1.49]). Those who reported a monthly household income ≤ $300 evidenced 1.75 higher odds of being diagnosed compared to patients with incomes ≥ $2101 per month (OR: 1.75, CI [1.25, 2.46]). Patients with a previous mental disorder had 1.90 higher odds of receiving a neurocognitive diagnosis compared to patients without a previous illness (OR: 1.90, CI [1.57, 2.31]). Employment status, level of education, and history of substance use/abuse was not significantly associated with being diagnosed with a current DSM-5 neurocognitive disorder. Further, after adjusting for multiple comparisons, all sociodemographic and previous mental disorders remained significant.

#### Anxiety disorders

Only age and recurring illness was significantly associated with being diagnosed with a current anxiety condition. For every one-year increase in age, patients had 0.2% lower odds of being diagnosed (OR: 0.989, CI [0.981, 0.997]). Having a previous mental disorder was related to 4.46 higher odds of receiving a current diagnosis (OR: 4.46, CI [3.47, 5.72]). Both age and previous mental disorder remained significantly linked to a current anxiety disorder diagnosis after adjusting for multiple comparisons.

#### Substance-related disorders

Each year increase in age was associated with 2.9% lower odds of being diagnosed with a current substance-related disorder (OR: 0.97, CI [0.96, 0.98]). Female patients evidenced 73.2% lower odds of being diagnosed compared to males (OR: 0.26, CI [0.18, 0.39]). Conversely, males evidenced 3.73 higher odds of being diagnosed with a substance-related disorder. Unmarried patients evidenced 1.90 higher odds of receiving a diagnosis compared to married patients (OR: 1.90, CI [1.34, 2.68]). Those who reported monthly household incomes of ≤ $300 were 2.76 times more likely to have been diagnosed compared to those with a monthly income of ≥ $2101 (OR: 2.76, CI [1.40, 5.44]). Having a previous mental disorder was associated with 2.86 higher odds of receiving a current diagnosis compared to not having a previous illness (OR: 2.86, CI [2.06, 3.97]). Lastly, those with a history of substance use/abuse were 16.98 times more likely to have been diagnosed with a current substance-related disorder compared to patients without substance use/abuse history (OR: 16.98, CI [10.48, 27.49]). Level of education and employment were not significantly associated with receiving a current DSM-5 substance-related diagnosis. All covariates remained significant after adjusting for multiple comparisons.

#### Schizophrenia spectrum disorders

Each one-year increase in age was associated with 2.8% less odds of being diagnosed with a current schizophrenia spectrum disorder (OR: 0.97, CI [0.96, 0.98]). Female patients were 60% less likely to have been diagnosed compared to males (OR: 0.39, CI [0.27, 0.56]). This is to say male patients evidenced 2.53 higher odds of being diagnosed with a schizophrenia spectrum disorder compared to females. Unmarried patients were 2.49 odds more likely to have been diagnosed compared to married patients (OR: 2.49, CI [1.66, 3.73]). Those with less than high school education evidenced 2.13 higher odds of being diagnosed (OR: 2.13, CI [1.31, 3.47]) compared to patients with postsecondary education attainment or beyond. Patients with monthly household incomes of ≤ $300 and $301–$900 had 4.28 and 3.05 higher odds, respectively, of being diagnosed compared to those with household incomes ≥ $2101 per month. Patients with a past history of mental disorders had 30.16 higher odds of receiving a diagnosis than patients without past mental disorder histories (OR = 30.20, CI [18.67, 48.72]). All covariates except less than high school education attainment and monthly household income of $301–$900 remained significant after adjusting for multiple comparisons.

## Discussion

After controlling for covariates in the multinomial model, spurious associations were discovered among anxiety, substance-related, and schizophrenia spectrum disorders. Covariates of major depressive and neurocognitive disorders, however, not only maintained significance, but increased in odds ratio from the unadjusted model. Thus, the following discussion will highlight select implications regarding major depressive and neurocognitive disorders diagnosed in inpatients receiving medical or surgical care in a general hospital. Overall, 53% of our sample of medical inpatients received a current DSM-5 mental disorder diagnosis. While seemingly high, it is comparable to other studies that have characterized mental illness prevalence among medical inpatients [5, 18, 33, 34, 46].

### Major depressive disorders

Major depressive disorder (MDD) is a mood disorder that negatively impacts affective, cognitive, physiological, and behavioral systems. Hallmark features of MDD are sadness, inability to enjoy things that were once pleasurable, and a myriad of signs and symptoms ranging from lack difficulty concentrating and insomnia, to fluctuation in weight and changes in eating patterns. In the severest form, patients with MDD may evidence recurring thoughts of death, suicidal ideation, and even suicide attempts [3]. A recent epidemiological study revealed that among non-institutionalized community members, MDD was slightly higher in Puerto Rican adults living on the island (9.9%) compared to the U.S. adult population (8.5%) [10]. Traditionally, among U.S. adults, MDD rates are higher in females compared to males, 8.7% and 5.3%, respectively, and is most prevalent in people aged 18 to 25. [26]. Sociodemographic factors such as low educational attainment, economic instability, and lack of social support are implicated in the etiology and course of MDD [4].

Traditionally, females are disproportionally affected by MDD [2, 23, 26, 28]. However, this was not the case in this sample of general hospital inpatients. Male patients evidenced 1.30 higher odds of being diagnosed with a major depressive disorder compared to females. At least two plausible explanations can account for this finding. First, in this setting where evaluations and diagnoses were made (a general hospital), both males and females were equally likely to have received a psychological evaluation and subsequent diagnosis, if warranted. Yet, this is not the case in community and outpatient settings, where females are more likely to seek mental healthcare services than males [25, 36, 38]. It begs the question if higher MDD prevalence rates among women are a function of healthcare-seeking behavior or due to actual biological sex-related difference in development and course of depression. Secondly, considering that male patients evidenced higher proportions of mental and physical illness comorbidities than females, 59% and 48%, respectively, it is plausible that males engaged in relatively maladaptive coping mechanisms in managing their conditions, contributing to depressive affect and subsequent diagnosis during hospitalization. Nevertheless, this does not fully explain why male patients evidenced higher odds of being diagnosed with MDD during hospitalization compared to females.

Regarding the associations between sociodemographic variables spanning age, civil status, employment status, income level and a current diagnosis of depression among this present study’s sample, our findings are consistent with existing literature describing non-medical and medical populations [1, 4, 27, 32, 33, 46]. In short, relatively older age and being married is associated with a lower incidence of receiving a current depression diagnosis. Unemployment (including retirement), and lower income is associated with higher incidence of receiving a current depression diagnosis (see Additional file 1).

### Neurocognitive disorders

Neurocognitive Disorders (NCD) are a group of disorders that are characterized by significant clinical dysfunction in areas of complex attention, executive functioning, perpetual motor skills, social cognition, and emotional reaction. Such dysfunction is readily recognizable from premorbid functioning and unfolds in a progressive manner [3]. Considering there are 10 million new cases of NCDs registered yearly, it is estimated that by 2030 there will be an alarming 82 million people suffering from a form of NCD worldwide [47]. NCDs have been among the most prevalent causes of death in the world. While much less is known about the prevalence rates of NCD in Puerto Rico, researchers have estimated that 5% of adults who are 45 years of age and older report cognitive decline. Of those 5%, the vast majority (87%) of people with subjective cognitive decline have at least one chronic physical condition [11].

In our sample, as expected, relatively older age was associated with receiving a current NCD diagnosis, specifically, a delirium diagnosis during hospitalization, where each one-year increase in age was associated with 1.05 higher odds of receiving an NCD diagnosis. Delirium disorders are common among elderly patients admitted to general hospitals, with estimates ranging from 14 and 56% of all elderly patients being affected during hospital stay [3, 16]. Even though most patients that present with delirium fully recover, if not detected and treated promptly, delirium can lead to adverse consequences and even mortality, especially among older patients [19]. Interestingly and fortunately for the patients, in this present study, unlike major depressive disorders, NCDs were diagnosed more owing to the standing order routine evaluations compared to consults requested by attending physicians, suggesting that if it had not been for routine the evaluations in place, rapid identification of an NCD and initiation of appropriate psychosocial interventions would have possibly been forfeited.

In our study, the second most single common NCD was unspecified neurocognitive disorder, accounting for 14.8% of all NCD diagnoses. In terms of sociodemographic factors, increasing age, especially individuals over 60 years is associated with receiving a current NCD diagnosis [3]. While there are sex-related differences associated with various subtypes of NCDs, generally, females have higher rates of diagnosed NCDs compared to males. This difference is believed to be due to greater longevity in females [17]. Our findings differ slightly from existing literature as males were more likely to have received a current NCD diagnosis. In fact, female patients evidenced 45% lower odds of being diagnosed with an NCD than males. We believe these findings are a function of the healthcare seeking behavior specific to this sample as discussed in the section regarding MDD diagnoses.

### Key implications

The vast majority (90%) of patients that were diagnosed with MDD and 87% that were diagnosed with NCD received an evaluation within 24 h of being admitted to corresponding hospital units or after physician or patient requested consultations. Swift detection of a current mental disorder during hospitalization and initiation of appropriate interventions is largely due to the integration of clinical health psychology services within a general hospital care delivery model. The CPSP is part of a process-based integration model [22]. In a process-based integration model, a set of methods and models are intertwined to ensure the alignment of financial, administrative, organizational, and clinical functions, with the intention of effectively combining the provision of two or more originally separate clinical services (eg. Coronary care and clinical health psychology). When done correctly, process-based integration results in a fluid, interdisciplinary approach that generates benefits for the whole person/patient and can be conceptualized as integrated care [22]. The implementation of process-based comprehensive care models can be located within one or more of the following general typologies: (a) organizational, (b) functional (c) service, and (d) clinical [24]. Organizational integration occurs when two or more separate organizations (for example, a general hospital and a graduate health sciences institution) join forces to some extent to align the delivery of care for a given patient population. Functional integration is the exchange of non-clinical support components, such as electronic medical records, within and between organizations and clinical units. Service integration is most easily identified as a team of professionals offering their perspective clinical services in a comprehensive and interconnected manner. Finally, clinical integration requires the provision of care by an interdisciplinary team through the use of shared guidelines or protocols (for example, a standing administrative order requiring that all patients admitted to the clinical unit undergo a screening) and standardized assessment.

The model implemented by the CPSP incorporates, to some degree, organizational, functional, service, and clinical components. The program has two modalities of patient evaluation (standardized administrative orders and medical referrals/consults). There is organizational integration established through a collaborative agreement between Hospital Damas and Ponce Health Sciences University (the home institution of our clinical and research teams). Also, the CPSP is located in physical space within the hospital which allows for quick response times and direct contact with doctors and nurses. Contact with these health service providers helps in obtaining direct information on the medical conditions of patients, contributes to reaching an accurate diagnosis of mental disorders, and facilitates the implementation of comprehensive treatment. This approach has been shown to help improve health outcomes for patients and reduce costs related to healthcare expenses [12].The theoretical model of integrated care on which CPSP operates was designed and implemented by [20], the CPSP coordinator and senior researcher and author of our research team.

Interventions facilitated by the clinical team were implemented at the patient’s bedside during the course of the initial clinical evaluation. Follow-up sessions were scheduled if warranted. Interventions were tailored to meet the patient’s most pressing need during hospitalization and spanned cognitive and behavioral techniques primarily aimed at improving cognitive restructuring, emotion regulation, activation of physiologic relaxation response and solution focused techniques to enhance adherence to medical treatment regimens. While not detailed in this present study, the clinical team referred patients to specialty care services when necessary and implemented brief interventions with family and other healthcare workers including physicians, nurses and social workers as needed to enhance the clinical health psychology services provided. Additionally, patients that require out-patient behavioral health services (e.g., psychotherapy and pharmacotherapy) are referred to our community health center (Wellness Center of Ponce Health Sciences University).

### Strengths, limitations, and future work

While the results from this study cannot support a causative link between sociodemographic factors, previous psychiatric history, and current mental disorder diagnosis, the relatively large sample size and applied research approach allow these findings to be generalized outside of this sample of patients. A possible limitation for this study, however, is the research team did not analyze quantitative psychological symptom inventories (e.g., BDI-II) as covariates. These inventories are administered at patient’s bedside to aid in arriving at an accurate current diagnosis. However, these inventories were not available in the digital database as were the other variables analyzed in this study. Also, our clinical team did not implement measures to initiate patient follow-up post hospital discharge for symptom monitoring or continued psychotherapy. Due to the brief (average two-to-three sessions) and often time single session encounters that is common in C-L psychology in general hospital settings, it is challenging to gauge the effectiveness of the treatment interventions once patients are discharged. This is concerning as the ultimate goal of intervening in medical patients is to enhance overall health outcomes by addressing pressing mental health challenges that may be interfering with physical health and daily functioning. When there is no follow-up system in place, there is no way of knowing if patients actually benefited from the interventions of if they require more long-term therapy. There is limited literature detailing the effectiveness of post-discharge C-L follow-up systems in identifying areas of patient need and continued therapy [13, 35]. Thus, future clinical and research work related to designing and implementing post-discharge follow-up is a promising avenue.

## Conclusion

Major depressive, neurocognitive, anxiety, substance use, and schizophrenia-spectrum disorders were the five most commonly made DSM-5 diagnoses in a general hospital system. Male patients were 1.30 times more likely to have been diagnosed with major depressive disorder compared to females, and delirium was the most common form of NCD among both males and females. The importance of integrating clinical health psychology services within the administrative and clinical structure of general hospitals was discussed. Future work concerning the opportunities, barriers and program evaluation methods are needed. Additionally, ways of continuing and or monitoring of psychological care post discharge is worth future clinical and research efforts.

## Supplementary Information

**Additional file 1.** Commonly Diagnosed Mental Disorders in a General Hospital System.

## Data Availability

Not applicable.
