# Supplementary material for: Commonly diagnosed mental disorders in a general hospital system
Source: Int J Ment Health Syst. 2021 Jun 19;15:61. doi: 10.1186/s13033-021-00484-w (PMC8214275; doi:10.1186/s13033-021-00484-w)
Supplement: Supplementary file 1 — Additional file 1. Commonly Diagnosed Mental Disorders in a General Hospital System. [file 13033_2021_484_MOESM1_ESM.docx]

JMHS-D-20-00229
Commonly Diagnosed Mental Disorders in a General Hospital System

Reviewer’s Comments Table

2^nd^ Revision: April 7, 2021

| **Manuscript Revision Table** | | |
| --- | --- | --- |
| Reviewer | Comment | Authors’ response/action |
| 2 | My only outstanding comment is that the Abstract should focus on other variables entered into the model, as only presenting gender could be perceived as selective reporting. This does not have to add much to the length of the Abstract and could easily be done within the world limit. | As suggested, we added a section mentioning the other sociodemographic variables in relation to the current DSM-5 diagnoses. |
| 3 | I think the authors should also summarize the prevalence of the "Chronic Co-morbid Physical Illness" in table 2 for the entire cohort as well. | As suggested, we’ve summarized these conditions. |
| 3 | “If the participants were enrolled because of physical illness rather than mental illness …Therefore, when describing the co-morbid chronic physical conditions by mental disorders in Section 3.2, instead of saying "the most and least frequent physical conditions for major depressive disorder are XYZ," the authors should frame it as "24% of the patients with cardiovascular diseases had major depressive disorder”. | Thank you for suggesting this modification. We would like to clarify that patients of our sample were not enrolled because of their physical condition. As we’ve detailed in the methods section, our program offers psychology consultation-liaison services to patients admitted to select clinical units of a general hospital. And the focus of this manuscript is not physical conditions, rather mental illness. We realize that by changing the semantics of how this section is written to a physical illness focus will not necessarily change the findings. However, we also realize that by leaving it how it is written will not change the findings either. It will, however, change the focus and theme of our manuscript: mental illness. By maintaining this phrasing and focus we will keep readers engaged in the mental illness theme. |
| 3 | For the two-direction interpretation of odds ratios (page 11 lines 31-32), if the authors really think male vs. female is more important than female vs. male, then treat female as the reference group and report and only interpret the odds ratios for male vs. female. The audience should be able to do the reversed interpretation. | Thank you for bringing this to our attention again. We believe this point is one of differing styles and not of scientific rigor. Reporting the ORs and providing a two-direction interpretation does not alter the findings or message in any fundamental way. Besides, providing a two-direction interpretation of ORs is a common practice observed in the literature and we believe is appropriate as we are careful to not assume that every person that reads our manuscript will have a working understanding of ORs and how to interpret them. To maintain clarity, we believe our two-direction interpretation is in order. |
| 3 | If the authors applied the Benjamini-Hockberg procedure to control for false discovery for multiple comparisons, why did the authors present and interpret the (the majority of the) results based on the p-values before the adjustment? | Thank you for bringing this to our attention. So, when using the Benjamini-Hockberg procedure the adjusted p-values are rank-ordered and all original p-values that are less than the adjusted p-values are reported as maintaining significance after controlling for multiple comparisons. In our case, all original p-values were less than the adjusted p-values, so there is no need to report the adjusted values. It is customary to report the adjusted p-value when using the Bonferroni method as there is only one adjusted p-value and there is no rank-ordering of those values, as there is with the Benjamini-Hockberg procedure. |
